# Root Foraging Increases Performance of the Clonal Plant *Potentilla reptans* in Heterogeneous Nutrient Environments

**DOI:** 10.1371/journal.pone.0058602

**Published:** 2013-03-05

**Authors:** Zhengwen Wang, Mark van Kleunen, Heinjo J. During, Marinus J. A. Werger

**Affiliations:** 1 State Key Laboratory of Forest and Soil Ecology, Institute of Applied Ecology, Chinese Academy of Sciences, Shenyang, China; 2 Ecology and Biodiversity Group, Institute of Environmental Biology, Utrecht University, Utrecht, The Netherlands; 3 Institute of Plant Sciences, University of Bern, Bern, Switzerland; 4 Ecology, Department of Biology, University of Konstanz, Konstanz, Germany; University of Alberta, Canada

## Abstract

**Background:**

Plastic root-foraging responses have been widely recognized as an important strategy for plants to explore heterogeneously distributed resources. However, the benefits and costs of root foraging have received little attention.

**Methodology/Principal Findings:**

In a greenhouse experiment, we grew pairs of connected ramets of 22 genotypes of the stoloniferous plant *Potentilla reptans* in paired pots, between which the contrast in nutrient availability was set as null, medium and high, but with the total nutrient amount kept the same. We calculated root-foraging intensity of each individual ramet pair as the difference in root mass between paired ramets divided by the total root mass. For each genotype, we then calculated root-foraging ability as the slope of the regression of root-foraging intensity against patch contrast. For all genotypes, root-foraging intensity increased with patch contrast and the total biomass and number of offspring ramets were lowest at high patch contrast. Among genotypes, root-foraging intensity was positively related to production of offspring ramets and biomass in the high patch-contrast treatment, which indicates an evolutionary benefit of root foraging in heterogeneous environments. However, we found no significant evidence that the ability of plastic foraging imposes costs under homogeneous conditions (i.e. when foraging is not needed).

**Conclusions/Significance:**

Our results show that plants of *P. reptans* adjust their root-foraging intensity according to patch contrast. Moreover, the results show that the root foraging has an evolutionary advantage in heterogeneous environments, while costs of having the ability of plastic root foraging were absent or very small.

## Introduction

In most natural and semi-natural plant communities, root competition is ubiquitous and a major component of inter-plant interactions [1]. One of the features, besides physiological adjustments, that could confer competitive ability to plants is root foraging by means of plastic adjustments of root allocation and architecture [2]–[4]. Such plastic root-foraging responses have been widely recognized as an important strategy for plants to explore resources that are heterogeneously distributed both in space [5], [6] and in time [7], and thus may contribute to plant performance.

Soil nutrients in natural environments are distributed heterogeneously at spatial scales relevant to an individual plant root and to entire ecosystems [8], [9]. Consequently, most individual plants are likely to experience spatial heterogeneity in nutrient availability, and this is especially likely for horizontally spreading clonal plants, which dominate in many ecosystems [10]. A major aspect of spatial heterogeneity is patch contrast, which is defined as the relative difference in resource availability between patches [11]. Unless the background nutrient availability is so high that nutrients are not limiting plant growth, root-foraging responses are expected to increase with patch contrast [12].

If root foraging is very effective in allowing the plant to find and exploit high-resource patches, individual plants or plant assemblages in heterogeneous environments might perform better than the ones in homogeneous environments with the same total resource availability [13]–[17]. On the other hand, if root-foraging intensity is not optimal, and is associated with increased costs of resource transport, plants in heterogeneous environments might perform worse than the ones in homogeneous environments. A meta-analysis by Kembel & Cahill showed that, overall, performance of plants was slightly higher in heterogeneous than in homogeneous environments, but that there are also many species that have lower performance in heterogeneous than in homogeneous environments [18]. Even when plants perform worse in heterogeneous than in homogeneous environments, a high root-foraging intensity could still have improved their performance (i.e. reduced the negative effects) in heterogeneous environments. However, to the best of our knowledge, no study has tested explicitly whether performance of genotypes in a heterogeneous environment increases with root-foraging intensity, and how this depends on patch contrast.

Root foraging is one of the many forms of phenotypic plasticity exhibited by plants [18]. Although many studies have focused on the potential benefits of phenotypic plasticity, the potential costs of phenotypic plasticity have received much less attention [19]–[21]. Potential costs include, among others, the costs of maintaining the sensory and regulatory machinery required for plasticity and a less stable development [19]–[21]. Researchers have previously explored benefits and costs of plasticity in shoot characteristics such as branching frequency, stolon internode length and leaf length [22]–[26], but only few studies have addressed benefits and costs of plasticity in root characteristics. Fransen and de Kroon found that plastic root-foraging responses can have negative effects in the long term when the resource conditions have changed and the plastically induced phenotype is no longer adequate [27]. However, so far, potential costs of root-foraging ability (i.e. the ability of a genotype to plastically change its root-foraging intensity across environments of different heterogeneity) have received no attention [6].

To test the effect of patch contrast and directionality on root-foraging responses, and the benefits and costs of root foraging, we grew ramet pairs of 22 genotypes of the stoloniferous clonal plant *Potentilla reptans* at null, medium and high patch contrast. We asked the following specific questions: 1) Is root-foraging intensity positively correlated to the magnitude of patch contrast? 2) Do genotypes with a high root-foraging intensity have a higher performance than genotypes with a low root-foraging intensity when growing in heterogeneous environments? 3) Do genotypes with a strong root-foraging ability (i.e. plasticity in root-foraging intensity) perform worse than genotypes with a weak root-foraging ability when growing in a homogeneous environment (i.e. are there costs of having the ability for root-foraging when it is not needed)?

## Materials and Methods

### Plant Material

The experiment was carried out with *Potentilla reptans* L., a stoloniferous herb. Typically, the plant grows in moderately disturbed sites, productive pastures, mown grasslands, lake and river shores, road margins and other man-made habitats [28]. During the growing season, established ramets produce sympodial stolons, which may root and give rise to one daughter ramet at each node. The apical meristem develops into an embryonic flower, which may stay dormant or develop into a full-sized flower. The plant has been shown to have a high degree of clonal integration [29], [30], and thus can transport soil resources taken up by a ramet in a high-resource patch to connected ramets in low-resource patches. Note that although clonal plants like *P. reptans* can perform clonal foraging (i.e. selectively place ramets in certain patches by plastic changes in stolon-internode length and branching) [31], [32], we used the species here as a model for studying root foraging.

In the experiment, we used 22 genotypes of *P. reptans*, which originally had been collected at different sites in the Netherlands. No specific permits were required for the collection of plant material and for the described studies. Ten of the genotypes (coded as A–J alphabetically) had been kept in the Botanical Garden of Utrecht University, the Netherlands, since 1997 [4]. Another seven genotypes (Coded as K–Q alphabetically) were collected from the field in the early spring of 2009. The remaining five genotypes (Coded as R–V alphabetically) had been kept in a greenhouse of Nijmegen University, the Netherlands, since 2001 (Table S1).

### Pre-Cultivation and Experimental Setup

Pre-cultivation and the experiment were done in a partly controlled greenhouse with open sides (with 50% of daylight, while day length and air temperatures following approximately those outside). For pre-cultivation of ramet pairs, we transplanted individual ramets of each of the 22 genotypes into 2.5-L pots filled with compost (ZPV-0 type, Holland Potgrond BV, Wateringen, the Netherlands). We placed smaller 0.4-L pots filled with a 1∶1 mixture of compost and river sand around the original pots to receive the potential offspring ramets. Because the experiment was labor intensive, we performed the experiment in four replicate blocks that were started 1–2 weeks apart (June 27, July 13, July 20 and July 27, 2009). We explicitly accounted for variation among blocks in the statistical analyses (see the Data Analyses section below).

Each block comprised a whole set of 22 genotypes, with each genotype represented by six ramet pairs that were exposed to six treatments (three patch contrasts × two directions) as described below. To assure that the ramet pairs were of similar developmental stage, we always selected the 3rd and 4th ramet along a stolon (counting from the youngest one at the tip of the stolons) as an experimental ramet pair. We cut the selected ramet pairs off the rest of the clone with in-between stolon internodes left intact, and washed the roots free of soil very carefully. Altogether, we had 132 ramet pairs (six ramet pairs for each of the 22 genotypes) in each block (i.e. a total n of 528 ramet pairs). We standardized these ramet pairs for size by removing all the unfolded leaves, except for the two youngest ones, on each ramet and by cutting the roots to a length of 5 cm. As this was done for all ramet pairs, potential side effects of this damage should be the same for all ramet pairs.

For each of the four blocks, we transplanted the two ramets of each pair into two separate adjacent 1.6-L pots filled with a 2∶1 mixture of river sand and compost. We set up three resource-contrast treatments as uniform (Null patch-contrast treatment), intermediate (Medium patch-contrast treatment) and high contrast (High patch-contrast treatment) between the paired pots by adding slow-release fertilizer (8.4% NH_4_-N, 7.4% NO_3_-N, 11% P_2_O_5_ and 11% K_2_O; Osmocote Plus,Grace Sierra International, Heerlen, the Netherlands) to the two pots of each pair in such amounts that the nitrogen supply rate was 1.25, 0.75 and 0.25 g m^−2^ wk^−1^ for one pot and 1.25, 1.75 and 2.25 g m^−2^wk^−1^ for the other pot, for Null, Medium and High patch-contrast treatments, respectively. The total nutrient supply was thus the same for all three contrast treatments. For each block, we randomly assigned the six ramet pairs of each genotype to the three treatments, with two pairs for each.

**Figure 1:**
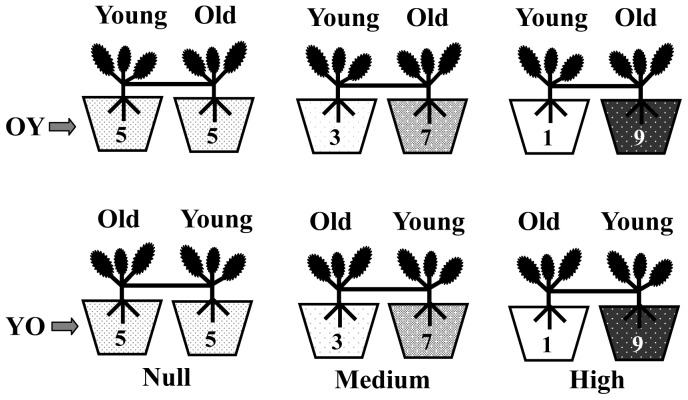
Schematic diagram of the experimental design for a genotype in each block. OY indicates that the ramet pair was transplanted in such a way that the developmentally older ramet was in the nutrient-rich pot, while the younger one was in the nutrient-poor pot. YO indicates that the ramet pair was transplanted in the reverse direction. Null, Medium and High indicate the three patch-contrast treatments. The numbers shown inside the pots indicate the relative nutrient availability.

When using ramet pairs, it is unavoidable that one is younger than the other. Previous studies have shown that, although resource translocation follows sink-source principles, natural resource translocation is predominantly from older to younger clone parts [33]–[37]. Moreover, the effects of integration on growth of the clone parts could depend on whether resource translocation is from old to young ramets or vice versa. Although the evidence for this is still limited [38], and it is hard to make predictions on how the direction of translocation should affect the allocation of resources to root growth of young versus root growth of old clone parts, it is important to account for such potential effects of directionality. Therefore, to allow for the detection of effects of directionality of ramet pairs, we transplanted one of the two ramet pairs of a genotype in each treatment in such a way that the developmentally older ramet was in the nutrient-rich pot, while the younger one was in the nutrient-poor pot (hereafter referred to as OY). We transplanted the other ramet pair in the opposite direction (hereafter referred to as YO; Fig.1). To avoid root foraging by new offspring ramets, we prevented the offspring ramets from rooting. We watered the pots when necessary.

### Measurements

We harvested all the plants in each block six weeks after they had been transplanted. Firstly, we counted the number of stolons, offspring ramets and flowers produced by each of the two originally planted ramets of each pair. Secondly, we cut all the aboveground parts, and separated them into original rosettes and stolons (including offspring rosettes). Thirdly, we washed the roots to remove soil. Finally, we weighed original rosettes, stolons and roots after drying them for >48hrs at 70°C.

### Data Analyses

We calculated patch contrast of each of the three treatments as the difference in nitrogen-supply rate between the patches divided by the sum of their nitrogen-supply rates. Similarly, we calculated root-foraging intensity for each ramet pair as the difference in root mass between ramets divided by the total root mass of the ramet pair. We calculated foraging ability of each genotype as the slope of the regression of root-foraging intensity of ramet pairs against patch contrast.

We used analysis of variance (ANOVA) to test the effects of patch contrast (Null, Medium, High), direction of ramet pairs (OY, YO), genotype and their interactions on root-foraging intensity, total biomass, number of offspring ramets and number of flowers. We accounted for variation among the four temporal blocks by including block as a factor in the model. Total biomass was ln-transformed to meet the assumption of homoscedasticity when performing ANOVA. For the calculation of correct *F* values, we considered patch contrast and direction as fixed factors, and block and genotypes as random factors (e.g. as error term of the patch-contrast effect, we used the patch contrast-by-genotype interactions instead of the residual). We also analysed number of offspring ramets and number of flowers as count data using generalized linear models and a Poisson distribution. However, because the results were very similar to the ones of ANOVA, we only present the results of the latter.

To test how root-foraging intensity depended on patch contrast (i.e. to test for root-foraging ability), we used linear regression separately for the two directionalities of ramet pairs. To get genotypic estimates of root-foraging ability, we also assessed the slopes of the regressions of root-foraging intensity on patch contrast for each genotype separately. To test for benefits of root foraging under different patch contrasts, we did regressions of fitness estimates (number of offspring ramets, number of flowers and total biomass) against root-foraging intensity realized in the Null, Medium and High contrast treatments. To test for costs of root-foraging ability, we did regressions of fitness estimates expressed in the Null contrast treatment against root-foraging ability of genotypes, which was assessed from the regression of root-foraging intensity on patch contrast for both directions of ramet pairs. To avoid bias in the selection gradients by environmentally induced covariation between root-foraging intensity and fitness, we used genotypic values instead of values of individual ramet pairs [39]. To allow for direct comparisons of regression coefficients, we expressed them in units of standard deviations, i.e., we used standardized regression coefficients [40]. All the statistical analyses were performed using the SAS program [41].

## Results

### Effects of Patch Contrast on Root Foraging and Fitness

**Figure 2:**
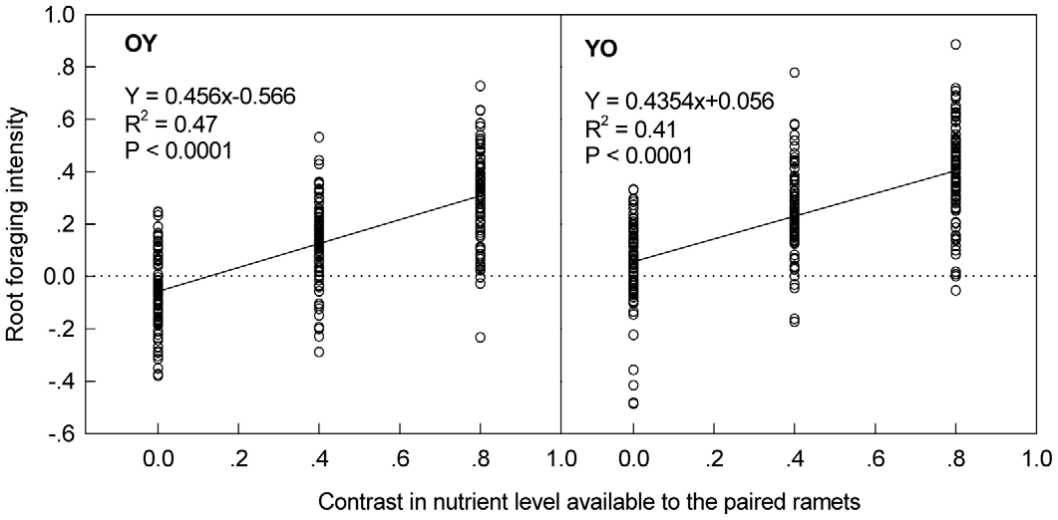
Dependence of root-foraging intensity of the ramet pairs on the contrast in nutrient availability. OY indicates that the ramet pair was transplanted in such a way that the developmentally older ramet was in the nutrient-rich pot, while the younger one was in the nutrient-poor pot. YO indicates that the ramet pair was transplanted in the reverse direction. Each dot represents a ramet pair.

Root-foraging intensity of ramet pairs was affected by patch contrast in nutrient availability (P<0.001) and direction of ramet pairs (P<0.001; Table 1). Root-foraging intensity was positively related to patch contrast, and was always greater when developmentally younger ramets were exposed to the higher nutrient level than the other way around (Table 1, Fig. 2). Although the slopes of the regression lines of root-foraging intensity on patch contrast were larger than zero, they were also smaller than one (P<0.001), indicating that root-foraging intensity was not fully proportional to patch contrast. Root-foraging intensity did not vary among genotypes, and the effects of patch contrast and direction on root foraging did not vary among genotypes either (Table 1).

**Table 1:** Results of three-way ANOVAs for effects of patch contrast, direction of nutrient gradient, genotype and their interactions on root-foraging intensity (RFI), total biomass, number of offspring ramets and number of flowers produced by the ramet pairs during the experiment.

Factors	d.f.	RFI		Total biomass		No. offspring ramets		No. flowers	
		*F*	*P*	*F*	*P*	*F*	*P*	*F*	*P*
Block	3	2.51	0.058	130.02	**<0.001**	84.42	**<0.001**	6.80	**0.002**
Contrast	2	187.21	**<0.001**	44.83	**<0.001**	21.15	**<0.001**	1.91	0.161
Direction	1	92.59	**<0.001**	5.53	**0.029**	2.56	0.125	0.48	0.495
Genotype	21	1.07	0.380	5.70	**<0.001**	16.14	**<0.001**	29.61	**<0.001**
C × D	2	0.50	0.610	0.40	0.672	0.09	0.912	0.34	0.715
C × G	42	0.83	0.761	0.79	0.825	0.75	0.876	1.65	**0.008**
D × G	21	038	0.995	0.43	0.987	0.29	0.997	0.29	0.999
C × D × G	42	0.80	0.805	0.59	0.982	0.49	0.997	0.64	0.962
Residual	393	0.033		0.066		583.707		25.320	

Values of P<0.05 are in bold. The residual mean squares are given in the bottom row

**Figure 3:**
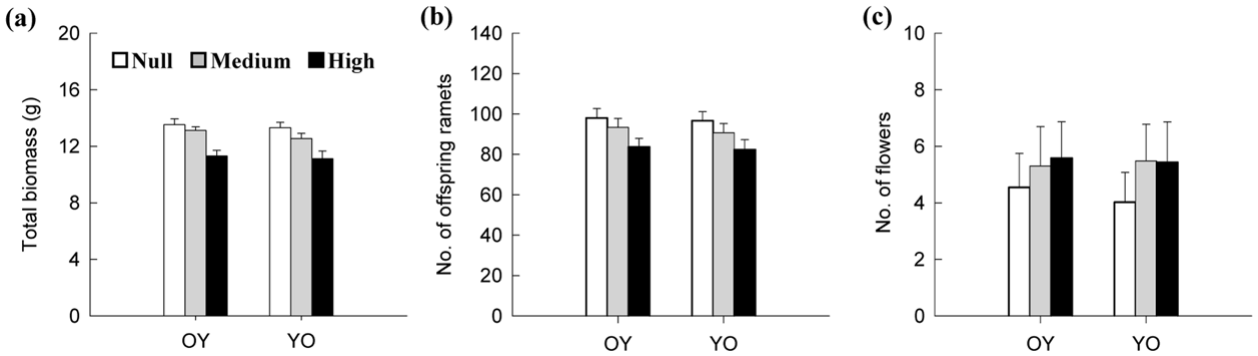
Comparison of fitness-related traits among ramet pairs under Null, Medium and High patch contrasts in nutrient availability. OY indicates that the ramet pair was transplanted in such a way that the developmentally older ramet was in the nutrient-rich pot, while the younger one was in the nutrient-poor pot. YO indicates that the ramet pair was transplanted in the reverse direction. For significance of the results, see Table 1.

Patch contrast affected total biomass (P<0.001) and number of offspring ramets (P<0.001), but showed little effect on the number of flowers. Direction had only an effect on total biomass (P = 0.029; Table 1). Total biomass and the number of offspring ramets were highest in the Null patch-contrast treatment and lowest in the High patch-contrast treatment (Fig. 3). On the other hand, the number of flowers was lower in the Null patch-contrast treatment than in the Medium and High patch-contrast treatments (Fig. 3c). All the three fitness estimates varied among genotypes (P<0.001), and also the effect of patch contrast on the number of flowers varied among genotypes (P = 0.008 for contrast x genotype interaction; Table 1).

### Benefits of Root Foraging

**Figure 4:**
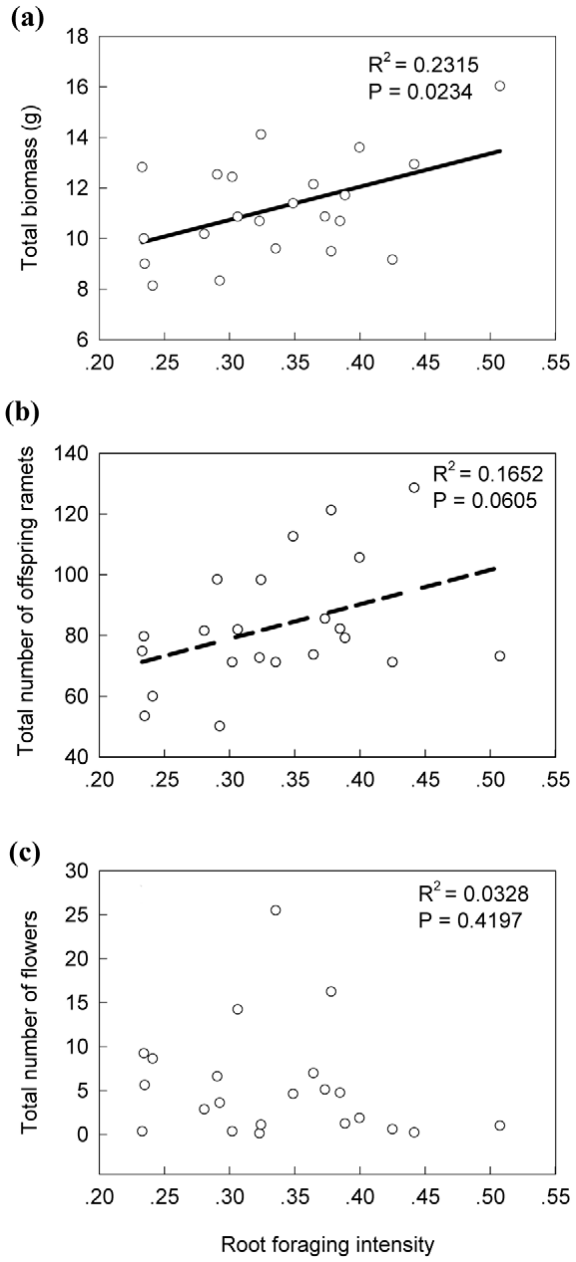
The contribution of root-foraging intensity to the fitness (A: total biomass; B: total number of offspring ramets; C: total number of flowers) of genotypes in the High patch-contrast treatment. The solid line in panel (A) and the dashed line in panel (B) show the significant and marginally significant regression lines, respectively. *R^2^* and *P* values for the regression lines are given in the upper-right corners of each panel.

In the Null contrast treatment, root-foraging intensity of genotypes did not affect any of the three fitness measures (Table 2). In the Medium contrast treatment, genotypes with a high root-foraging intensity tended to produce more offspring ramets (but not significantly so, P = 0.065), but not more biomass or flowers, than genotypes with a low root-foraging intensity (Table 2). In the High contrast treatment, genotypes with a high root-foraging intensity produced more biomass (P = 0.023), and tended to produce more offspring ramets (but not significantly so, P = 0.061), but not more flowers, than genotypes with a low root-foraging intensity (Table 2; Fig. 4). These results suggest that root foraging is beneficial in heterogeneous environments, and that it becomes more important with increasing patch contrast.

**Table 2:** Standardized regression coefficients ± standard errors and P-values for regression of genotypic values of fitness measures (total biomass, number of offspring ramets, number of flowers) on root-foraging intensity in treatments with different patch contrasts (Null, Medium, High).

Patch contrast	Total biomass		Number of offspring ramets		Number of flowers	
	Coefficient±SE	*P*	Coefficient±SE	*P*	Coefficient±SE	*P*
Null	−0.020±0.224	0.931	0.152±0.221	0.499	0.234±0.217	0.294
Medium	0.013±0.224	0.954	0.400±0.205	0.065	−0.004±0.224	0.987
High	0.481±0.196	**0.023**	0.406±0.204	0.061	−0.181±0.220	0.420

Values of P<0.05 are in bold.

### Costs of Root-Foraging Ability

**Figure 5:**
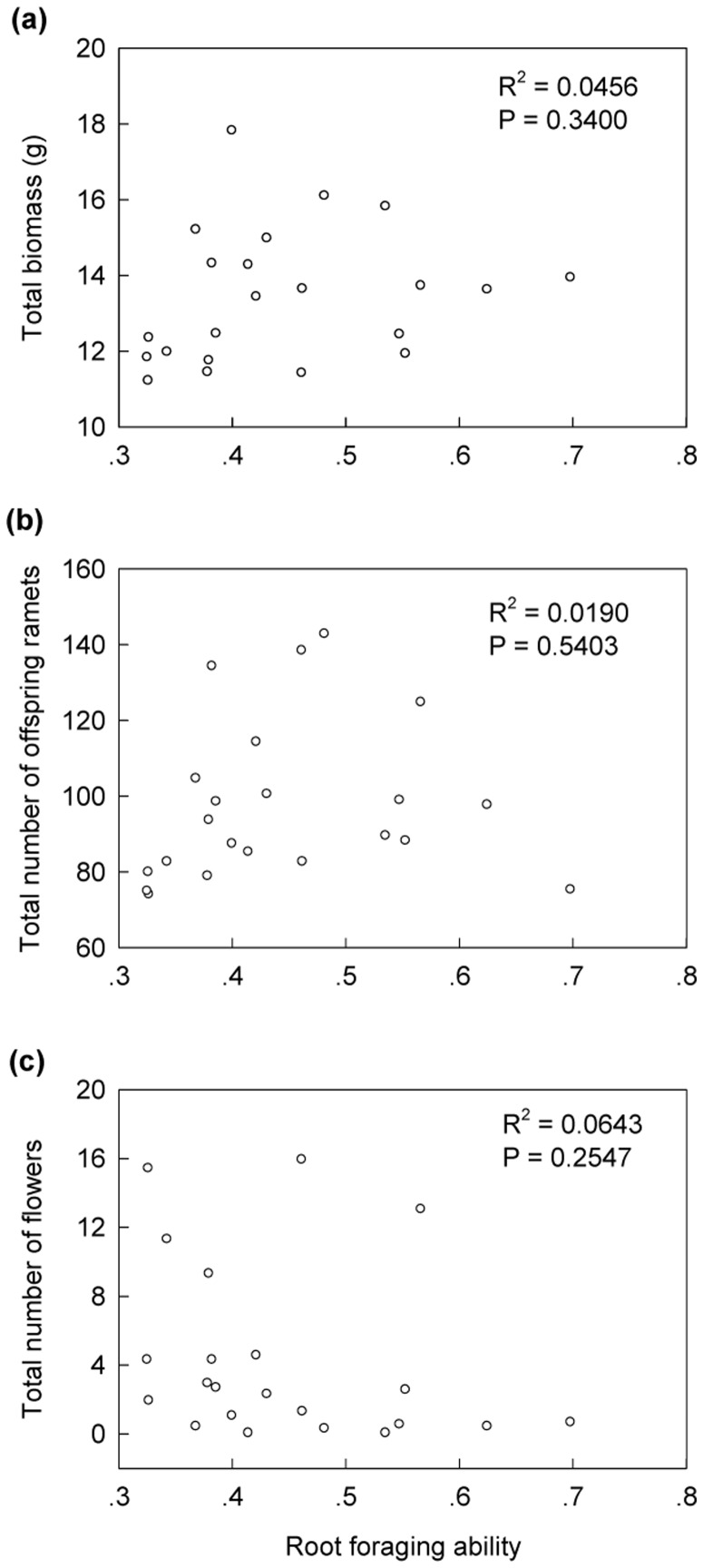
Tests for costs of root-foraging ability (defined as the slope of the regression of root-foraging intensity against contrast in nutrient level between the paired ramets). Fitness was measured as total biomass (A), total number of offspring ramets (B) and total number of flowers (C) in the uniform environment (i.e. the Null patch-contrast treatment).

Root-foraging ability of genotypes was not significantly related to any of the three fitness measures expressed when growing in the Null contrast treatment (Fig. 5).

## Discussion

Our study showed that despite the overall increase in root-foraging intensity with increasing nutrient heterogeneity (i.e. patch contrast), performance of ramet pairs of *P. reptans* was highest in the homogeneous treatment. Nevertheless, biomass production and vegetative reproduction of genotypes were positively correlated with their root-foraging intensity in the High-contrast treatment, indicating that root-foraging is beneficial in a highly heterogeneous environment.

### Relationship Between Root-Foraging Intensity and Patch Contrast

Root-foraging intensity increased with patch contrast, which is consistent with the prediction of a conceptual model by Lamb *et al.* [12]. Such a positive relationship was also evident in two empirical studies on *Glechoma hederacea* [14], [42]. In our case, plants adjusted their root allocation in such a way that nutrient acquisition per unit root became more equal between the connected ramets. This phenomenon is also a component of division of labor between interconnected ramets with regard to the functions of soil-resource uptake and photosynthesis [43]–[48]. In this sense, the theory of optimal allocation, which states that plants should adjust their allocation pattern in such a way that their growth is equally limited by all resources [5], [49], [50], may be expanded to include the situation in which the biomass allocation to plant organs capturing a certain resource is adjusted to be more proportional to the local abundance of this resource.

Although we did not have an *a priori* hypothesis for the effect of directionality, we found that root-foraging intensity was always greater when younger ramets were exposed to the higher nutrient level than when the older ramets were. This suggests that root plasticity is age-related in *P. reptans*. Root foraging is inherently coupled to physiological integration, which in clonal plants is frequently related to the direction of a ramet pair, and is usually acropetal [33]–[37]. However, this seems not to be the case in *P. reptans*, because resource transport in this species is more affected by source-sink relationships and not constrained by the direction of the ramet system [51], [52]. Possibly, the effect of directionality on root foraging can be explained by the greater sensitivity, plasticity, and growth rate of younger ramets.

### Benefits and Costs of Root Foraging

Regression of fitness-related traits against root-foraging intensity revealed that in the High patch-contrast treatment, genotypes with stronger root-foraging responses performed better. In the Null and Medium patch-contrast treatments, these relationships were not significant, although it was close to being significant for the analysis of number of offspring ramets in the Medium patch-contrast treatment. Probably, the contribution of root-foraging response to the fitness of the ramet pairs was only large enough to be detectable when there was a high patch contrast.

The reduced performance of ramet pairs under heterogeneous nutrient conditions suggests that there are current costs of root foraging responses and of associated processes such as the transformation of nutrients into transportable forms and nutrient transfer through the stolons [53]. Positive fitness effects of resource heterogeneity found in some previous studies suggest that such costs may be avoided or overcompensated by the selective placement of offspring ramets and roots in nutrient-rich patches [13], [54], [55]. In these previous studies, the observed benefits were most likely due to selective ramet placement rather than due to root-foraging responses. In our experiment, the two original ramets were intentionally transplanted into prescribed conditions, and subsequent foraging by selective placement of offspring ramets was experimentally prevented. Therefore, it is likely that, although the realized root-foraging responses were beneficial, they were not so strong that ramet pairs fully matched their root distribution to the pattern of resource supply [56]. Indeed, root-foraging intensity was not fully proportional to patch contrast (the slope of the regression in Fig. 2 is smaller than one). This indicates that overall resource uptake under heterogeneous conditions was lower than that under homogeneous conditions.

### Costs of Root-Foraging Ability

In addition to direct costs associated with root foraging, there is a risk that specialization of ramets becomes maladaptive (i.e. costly) when the stolon connection gets damaged [45], [57]. Moreover, there might also be costs of having the ability for root-foraging *per se*. Like for plasticity in any other trait, the costs of root-foraging ability may include *maintenance costs* for the sensory and regulatory machinery required for plasticity, *production costs* incurred when expressing a certain trait value over the costs that a canalized individual pays to express the same trait value, *information-acquisition costs* incurred to obtain environmental information, *developmental-instability costs* caused by suboptimal phenotype–environment matching due to environment-sensitive developmental course and *intrinsic genetic costs* as a result of pleiotropy, linkage or epistasis involving genes relevant for variation in fitness and plasticity [19]–[21], [58]. Such costs of plasticity have been proposed as explanation for why not all organisms have evolved perfect phenotypic plasticity [59], [60].

It has been proven very difficult to detect costs of plasticity [21], and in our study also no significant costs of root-foraging ability were detected. However, visual inspection of Fig. 5C suggests that there were two influential genotypes (A and B), without which the negative relationship between number of flowers in the homogeneous environment and foraging ability would have been significant. These two influential genotypes originated from nutrient-poor calcareous grassland habitats, and in a previous common-garden experiment, where ten of our genotypes (genotypes A to J, as used in the present experiment) were grown together at equal starting frequencies, they were the only ones that had disappeared from all plots after five years [4]. This suggests that the relationship between the number of flowers and the ability of plastic root foraging might be affected by the origin of the genotypes.

Several of the previous studies that found evidence for costs of plasticity, only found those costs when the plants were grown in stressful environments [22], [23], [61]. This suggests that costs of plasticity are more likely to be detected under resource limitation. If costs of root-foraging ability exist in our study system, the overall nutrient availability to the ramet pairs was apparently not low enough to allow detection of these costs. Therefore, future experiments on costs of root-foraging ability should not only use a patch-contrast gradient, but also establish a gradient of overall nutrient available to the whole ramet pairs.

## Conclusions

Our study clearly showed a positive relationship between root-foraging intensity and patch contrast. Thus, we suggest that optimal-allocation theory may be expanded by specifying that the biomass allocation to plant organs capturing a certain resource will be adjusted to be more proportional to the local abundance of the resource, so that these plant organs will be more equally limited. Our study further demonstrated clear benefits of root foraging in heterogeneous environments, in terms of biomass production and vegetative reproduction. However, we did not detect significant costs of having the capacity for plastic root foraging. Therefore, the question why root foraging has not evolved yet to such high levels that plants achieve equally high fitness in heterogeneous as in homogeneous environments remains unresolved. It would be worthwhile to further refine experimental set-ups, particularly by creating a finer and longer gradient in total nutrient availability. This will allow for a more precise assessment of evolutionary costs of root-foraging ability.

## Supporting Information

Table S1 **The origin of the 22 genotypes of** ***Potentilla reptans*** **used in the experiment.**
(DOC)
