# Supplementary material for: Root Foraging Increases Performance of the Clonal Plant Potentilla reptans in Heterogeneous Nutrient Environments
Source: PLoS One. 2013 Mar 5;8(3):e58602. doi: 10.1371/journal.pone.0058602 (PMC3589344; doi:10.1371/journal.pone.0058602)
Supplement: Table S1 — The origin of the 22 genotypes of Potentilla reptans used in the experiment. (DOC) [file pone.0058602.s001.doc]

Table S1

| **Table S1** The origin of the 22 genotypes of *Potentilla reptans* used in the experiment | | | |
| --- | --- | --- | --- |
| **Genotype** | **Origin place** | **Geographical Position** | **Habitat & Vegetation** |
| A | Eys, South Limburg, NL | 50°49’ N, 5°55’ E | Road side slope; annually mown chalk grassland vegetation |
| B | Klingeleberg, South Limburg, NL | 50°51’ N, 5°58’ E | Nature reserve; mown and grazed chalk grassland |
| C | Kwade Hoek Goeree, NL | 51°50’ N, 4°01’ E | Stabilized dune valley; annually mown, un-grazed grassland |
| D | Schalkwijk, Utrecht, NL | 52°00’ N, 5°11’ E | Road side verge; dense, nutrient-rich grassland |
| E | De Heul, Utrecht, NL | 52°00’ N, 5°07’ E | Disturbed recreation area; trampled grassland |
| F | Wijk bij Duurstede, Utrecht, NL | 51°58’ N, 5°19’ E | Road side verge; undisturbed vegetation with shrubs and grasses |
| G | Wijk bij Duurstede, Utrecht, NL | 51°58’ N, 5°19’ E | Car park on recreational area; heavily trampled grassland |
| H | Amerongen, Utrecht, NL | 51°59’ N, 5°27’ E | Road side verge; open and disturbed vegetation |
| I | Rhenen, Utrecht, NL | 51°57’ N, 5°37’ E | Open, sandy truck route (brick factory), no other vegetation |
| J | Achterberg, Utrecht, NL | 51°57’ N, 5°37’ E | Forest path; forest opening with tall grasses and forbs |
| K | Steenen Kamer nr Putten, Gld, NL | 52°16’ N, 5°32’ E | Mown grassland at the foot of riverside sand dune |
| L | Steenen Kamer nr Putten, Gld, NL | 52°16’ N, 5°32’ E | Roadverge (between farmland footpath and roadside ditch |
| M | Nieuwstad nr Elburg, Gld., NL | 52°27’ N, 5°50’ E | Edge of farmland (between farmland footpath and farmland) |
| N | Nieuwstad nr Elburg, Gld., NL | 52°27’ N, 5°50’ E | Edge of farmland (between farmland footpath and farmland) |
| O | Doornspijk nr Elburg, Gld., NL | 52°25’ N, 5°48’ E | Road verge; nearby roadside ditch |
| P | Veterinary Centre, Utrecht Univ., NL | 52°05’ N, 5°12’ E | Roadside (immediately next to road) |
| Q | Fort De Bilt, Utrecht, NL | 52°05’ N, 5°10’ E | In lawn beside the Botanical garden (next to the sidewalk) |
| R | Nijmegen, NL | 51°51’ N, 5°35’ E | In floodplain grasslands along the river Waal |
| S | Nijmegen, NL | 51°51’ N, 5°35’ E | In floodplain grasslands along the river Waal |
| T | Nijmegen, NL | 51°51’ N, 5°35’ E | In floodplain grasslands along the river Waal |
| U | Nijmegen, NL | 51°51’ N, 5°35’ E | In floodplain grasslands along the river Waal |
| V | Nijmegen, NL | 51°51’ N, 5°35’ E | In floodplain grasslands along the river Waal |
